# Implementation of pre-seasonal sublingual immunotherapy with a five-grass pollen tablet during optimal dosage assessment

**DOI:** 10.1111/j.1365-2222.2008.03153.x

**Published:** 2009-03

**Authors:** F Horak, S Jaeger, M Worm, M Melac, A Didier

**Affiliations:** * Medical University of Vienna, Department of ENT, Vienna, Austria,; † Department of Dermatology and Allergy, Charité Universitätsmedizin, Berlin, Germany,; ‡ Stallergenes, Medical Department, Antony, France; § Larrey Hospital, Respiratory Diseases Department, Toulouse, France

**Keywords:** allergic rhinoconjunctivitis, grass pollen allergy, optimal dosage, pollen peak, pollen season, pre-seasonal treatment, sublingual immunotherapy, sublingual tablet

## Abstract

**Summary:**

**Background:**

The optimal dose (300IR) of a five-grass pollen sublingual immunotherapy tablet in terms of efficacy was previously demonstrated from the first pollen season.

**Objective:**

Here, we aim to confirm whether this dose remained optimal during the peak of the pollen season by assessing the efficacy and quality of life data.

**Methods:**

A total of 628 subjects with grass pollen rhinoconjunctivitis were randomized in a double-blind, placebo-controlled, multi-centre, pan-European trial. Subjects received once-daily tablets (Stallergenes, Antony, France) of 100IR, 300IR, 500IR or placebo, starting 4 months before and throughout the 2005 grass pollen season. The pollen season was defined as the first day of 3 consecutive days with a grass pollen count above 30 grains/m^3^ of air, recorded using Hirst-type volumetric pollen traps, to the last day before 3 consecutive days with a pollen count below 30 grains/m^3^.

**Results:**

The grass pollen season lasted an average of 30 days, with a peak of 12 days. The mean treatment duration before the grass pollen season was similar in the four treatment groups (121.4±31.1 to 128.6±15.4 days in the safety population). Both the 300IR and 500IR groups had highly significant improvements in Rhinoconjunctivitis Total Symptom Score (RTSS) vs. placebo at the peak pollen season (*P*=0.0005 and 0.0014, respectively), which agreed with improvements in RTSS in the primary evaluations. The average RTSS scores were slightly elevated during the peak pollen season in all treatment groups. The overall Rhinoconjunctivitis Quality of Life Questionnaire score confirmed the optimal dosage 300IR at peak (*P*<0.0001) and at the end (*P*⩽0.0031) of the pollen season. All doses were well tolerated.

**Conclusion:**

At the peak pollen season, the efficacy and quality of life data for both 300IR and 500IR groups was significantly improved vs. the placebo group. These results confirm the conclusions of the primary evaluations and validate the use of 300IR tablets for clinical practice.

## Introduction

Prevalence of respiratory allergic diseases in Europe has been on the increase in the past decades [1]. Allergic rhinitis is a global health problem that affects 30% of the population world-wide. The prevalence of pollen allergy is presently estimated to be up 40%, and grass pollen is the major cause of pollinosis in many parts of the world [2]. Using the European Community Health Respiratory Survey I data, the geographical variation in sensitization to environmental allergen was recently measured and published. Sensitization was most common for allergens *Dermatophagoides pteronyssinus* (21.7%), grass pollen (16.9%) and cat (8.8%) [3].

Inhalation of pollen grains induces respiratory allergy symptoms in sensitized individuals, and the characteristic of seasonal allergy is the recurrent occurrence of symptoms. Patients experience symptoms only during the circulation season of aeroallergens to which they are sensitive [4]. Grass allergens induce mostly nasal and conjunctival symptoms and natural exposure to grass pollen may exacerbate asthma by inducing an inflammatory response (T cells, mast cells, eosinophils) [2].

The concentration of airborne grass pollen influences the degree of symptoms in pollinosis patients. The atmospheric concentration of grass pollen able to induce respiratory symptoms was shown to be 10–50 grains/m^3^ in London, 37 grains/m^3^ in Bilbao and 30 grains/m^3^ in Finland [2]. In Europe, the main pollination period covers about half the year, from spring to autumn. The main grass flowering period starts at the beginning of May and finishes at the end of July. In the Mediterranean area, flowering usually starts and ends 1 month earlier. Pollination occurs about 2–3 weeks earlier at sea level than in mountainous regions. The pollen season tends to vary from year to year because of fluctuations in climatic factors, but the maximum atmospheric concentration of grass pollen usually occurs 1–2 months after the start of the main flowering season. On the whole, in Europe, grass flowering notoriously peaks in June.

In a recent meta-analysis, the sublingual immunotherapy (SLIT) represents an effective treatment for allergic rhinitis in adults [5]. This has been corroborated by a meta-analysis of 22 controlled clinical trials including 979 adult patients with allergic rhinitis, which demonstrated that SLIT was significantly more efficacious than placebo [6]. However, the methodology of many specific immunotherapy trials was found to be insufficient until recent large pivotal studies [7–9]. Based on recognized efficacy measures, the current study has shown that the 300IR SLIT tablet containing five grass pollens was well tolerated and effective in reducing the symptoms of rhinitis and conjunctivitis and represents the optimal dose [9].

The purpose of this paper is to present the methodology used in a European study, which took into consideration the variability in the pollen season at different centres to obtain the same duration of treatment across the study. The primary objective of this study was to determine the optimal dose of five-grass pollen SLIT tablet, which has previously been reported [9].

## Materials and methods

This study was a randomized, double-blind, placebo-controlled trial conducted in Europe.

### Subjects

Adult subjects (18–45 years) with moderate to severe seasonal grass pollen-allergic rhinoconjunctivitis for at least 2 years (confirmed by a positive skin prick test and serum-specific IgE of at least Class 2) and with a score of at least 12 on the Retrospective Rhinoconjunctivitis Total Symptom Score (RRTSS) were enrolled. Eligible subjects were randomized 1 : 1 : 1 : 1 to one of the four treatment groups (100IR, 300IR or 500IR doses of a five-grass pollen SLIT tablet, or placebo tablet) using a computer-generated randomization list. Treatment began 4 months before the expected start of the pollen season in the area of the individual trial centre.

### Five-grass pollen tablets

The SLIT tablets contained standardized lyophilized allergen vaccines of five grasses: orchard or cocksfoot (*Dactylis glomerata*), meadow (*Poa pratensis*), perennial rye (*Lolium perenne*), sweet vernal (*Anthoxanthum odoratum*) and timothy (*Phleum pratense*) grasses. Tablet formulations are more stable, allow more precise dosage and are easier to take than liquid formulations [10, 11].

Subjects were given one of the three daily doses (100IR, 300IR or 500IR) of the SLIT tablets [IR is a measure of biological potency (skin reactivity) used to describe the strength of an allergen extract]. The mean dosage of 300IR/mL corresponded to approximately 25 mg/mL of the group 5 major allergens. Such a five-grass pollen extract represents natural exposure and sensitization conditions encountered by grass pollen-allergic subjects in Europe [12]. Allergen or placebo tablets were taken sublingually once daily in the morning before eating or drinking. The subject was instructed to keep the tablet under the tongue until complete dissolution before swallowing.

During the first 5 days of treatment, 100IR incremental increases in dose were administered until the final dose was achieved. To maintain the blinding, subjects took two tablets per day during the first 5 days of titration and one tablet per day from Day 6 until the end of treatment. For each patient, eight visits were scheduled: screening, randomization, 2 weeks after randomization, 4 weeks before the estimated start of the pollen, at the peak and at the end of the pollen season, at the end of treatment and a follow-up visit 2 weeks later. Subjects were enrolled in the study from 16 December 2004, and the final patient visit was on 5 September 2005.

### Pollen counts

Pollen traps closed by the individual investigators' centres were identified for the pollen counts. Data collected over the past 10 years (1997–2006) by the European Aeroallergen Network (EAN) from 497 monitoring sites was used to estimate the expected start date of the pollen season at each study investigational centre, which enabled treatment initiation 4 months before the start of the season.

As an arbitrary threshold for symptoms in the majority of sensitized subjects, a daily mean of 30 pollen grains/m^3^ of air has been chosen. The threshold of 30 for grass pollen has been frequently used in other multi-centre studies and may therefore be considered as consistent with other analogous studies. Allergologists in all the study sites have confirmed that the proposed definition of the grass pollen season coincided almost exactly with periods when their subjects were symptomatic.

- *Pollen estimation before the study*: For estimation of start, peak and end of the grass pollen season in different European regions, historical data were used from Hirst-type volumetric pollen traps. The average daily counts over several years have been displayed as a smoothed curve. Start dates have been calculated as the first day with a grass pollen count of 30 grains/m^3^ or above, and the end date was defined as the last day with this value. Pollen curves were available for each site.
- *Pollen season definition during/after the study*: Daily pollen counts for every site included were available during the study and retrospectively. The start, end, duration and intensity of the season of each site were defined. All data on study centre-specific pollen seasons was collected by the EAN.
- *Methodology of pollen sampling*: All monitoring stations involved follow the methodology recommended by the International Association for Aerobiology and the European Academy of Allergology and Clinical Immunology: the flow rate of the sampler is 10 L/min, sampling height is about 15 m above ground level, adhesives are either Vaseline or silicone fluid, 2 hourly pollen counts are added to one daily value that is expressed as daily average in pollen/m^3^ of air.
- *Definition of the pollen season and peak pollen season*: The pollen season was defined as the first day of 3 consecutive days with a grass pollen count above 30 grass pollen grains/m^3^ of air to the last day before 3 consecutive days with a pollen count under 30 grains/m^3^. Peak pollen period was defined as the period of 14 days with the highest grass pollen count in the area.

### Primary and secondary evaluation criteria

The primary efficacy assessment of the study was the RTSS, which was a combined assessment of the severity of the six rhinoconjunctivitis symptoms (sneezing, rhinorrhoea, nasal pruritus, nasal congestion, ocular pruritus and watery eyes) during the previous 24 h, and the results of this outcome have been reported elsewhere [9]. From approximately a month before, and during, the pollen season, subjects completed a daily card to score nasal and ocular symptoms, using the RTSS.

Secondary assessments were the individual rhinoconjunctivitis symptom scores and the RTSS at the peak of the pollen season. Subjects' quality of life was assessed using the self-administered disease-specific Rhinoconjunctivitis Quality of Life Questionnaire (RQLQ) [13]. Furthermore, subjects' evaluation of overall effectiveness was categorized as treatment success (score of 2=slight to moderate improvement or 3=good to excellent improvement in their condition) or treatment non-success (score of 0=worsening or 1=no change in their condition). In case of severe symptoms, subjects could use rescue medication. Subjects were instructed to start with an oral antihistamine (cetirizine) and if the symptoms were not alleviated, progress to an intra-nasal corticosteroid (mometasone furoate). Thus, the proportion of days with and without rescue medication could be calculated and compared between treatment groups.

Immunological parameters (grass –allergen-specific IgE and IgG4) were measured in serum samples taken from participants before and after treatment, as described by Didier et al. [9].

Safety data were analysed descriptively. All patients who received at least one dose of the investigational product were included in the safety analysis.

### Statistical methods

The primary analyses were done for the intent-to-treat (ITT) and per protocol (PP) populations. The secondary analyses were done for the ITT population alone.

Because the data were skewed, non-parametric methods were also used on the primary efficacy variable as a supportive analysis. All data are given as means±SD and medians. For the primary outcome, the difference between each active and placebo group was estimated by the difference in adjusted means together with the 95% confidence interval (CI) for this difference.

IgE and IgG4 levels for specific grass pollens were log-transformed and presented as geometric mean and 95% CI. Changes from baseline were expressed as ratios: after/before treatment.

Proportion of days with rescue medication, calculated during the pollen season, was analysed using Wilcoxon' two-sample test.

## Results

### Subjects

In total, 749 subjects were screened, of whom 628 subjects were randomized to the study and validated for inclusion in the safety protocol. Fifty-nine subjects were excluded from the ITT population, because their RTSS was unavailable for the pollen season, and 104 subjects were excluded from the PP analysis due to poor compliance, use of prohibited medications, and unavailability of RTSS, or withdrawal for a reason other than adverse event or lack of efficacy. Of the 628 randomized subjects, a total of 569 subjects comprised the ITT population, 559 of whom completed the study. Primary reasons for withdrawal were the following: lost to follow-up (11), withdrawal of consent (33), adverse event deemed to be severe enough by the investigator (17). These 17 adverse events led to the discontinuation of the study treatment in three (100IR group), six (300IR group) and eight (500IR group) of the patients. These events are considered moderate to severe by the investigator. One of them (pregnancy) is not related to the treatment. The other adverse events are local pain (4), oral pruritus (3), oropharyngeal swelling (2), dysphagia (1), oesophagitis (1), localized urticaria (1), rhinitis (2), cough (1) and atopic dermatitis (1). None of these adverse events was reported as a serious adverse event. The overall participant flow for the study is as described by Didier et al. [9]. The baseline demographic and clinical characteristics of each group were similar as was the mean treatment duration before the pollen season (121.4±31.1 to 128.6±15.4 days in the safety population). The mean duration of treatment was consistent across all centres involved in the study.

### Pollen season

The mean duration of the pollen season 2005 was 29.5±9.5 days and the worst pollen period 12 days. Of the 42 sites that randomized patients, the Czech sites had the longest pollen period (22–44 days) and the Hungarian sites had the shortest pollen period (8–36 days).

**Figure 1:**
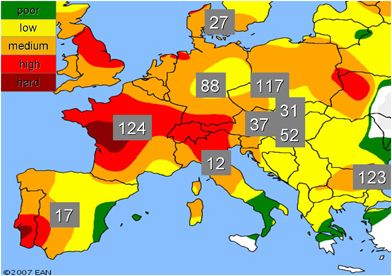
Levels of pollen exposure at the different study centre sites.Numbers in grey boxes refer to the number of subjects recruited at each study centre.

**Figure 2:**
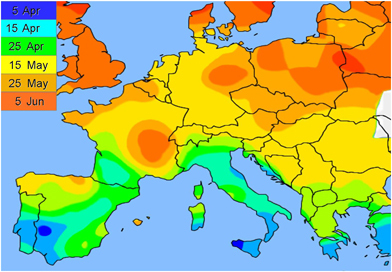
Variations in the beginning of the grass pollen season across Europe.Dates shown indicate the start date of the pollen season in each study centre.

**Figure 3:**
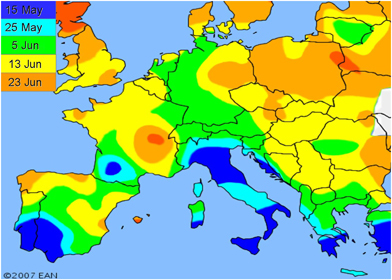
Variations in the timing of the peak pollen season across Europe.Dates shown are indicative of beginning of the pollen peak for each study centre.

The relative intensity of grass pollen seasons in various areas was obtained by plotting the average cumulative grass pollen counts (Fig. 1). The variation in dates at which the grass pollen season began across the different study centres is shown in Fig. 2. As a general rule, the grass pollen season starts earlier in the south of Europe than in the north. As an indicator of the peak season, the median of the peak season has been chosen (50% of the annual cumulative grass pollen count – with 25% of the annual total cut-off at both ends). The map in Fig. 3 shows the median peak, day distribution during an average grass pollen season.

### Efficacy of treatment

**Figure 4:**
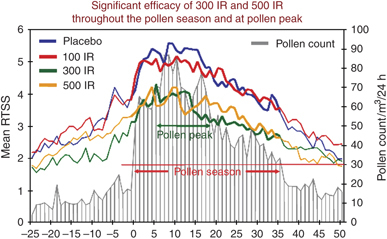
Efficacy outcomes: main criteria Rhinoconjunctivitis Total Symptom Score (RTSS) showing significant efficacy of 300IR and 500IR tablets throughout the pollen season at pollen peak.

The effect of treatment, assessed by the mean difference in RTSS between each active group and the placebo group (including the 95% CI), was highly significant for the 300IR [−1.39 (−2.09; −0.69), *P*=0.0001] and 500IR groups [−1.22 (−1.91; −0.53), *P*=0.0006] but not significant for the 100IR group [−0.26 (−0.95; 0.43), *P*=0.46]. Consistently both 300IR and 500IR showed a highly significant difference in RTSS compared with placebo during the pollen season (*P*=0.0005) and also at peak pollen season (*P*=0.0014). The average RTSS scores were slightly higher during the worst pollen period in all treatment groups. Comparisons of efficacy in the active groups vs. the placebo group using only the 10 pooled sites with the highest pollen counts showed statistically significant differences between the 300IR and 500IR groups vs. the placebo group, but no statistically significant difference between the 100IR group vs. placebo (Fig. 4).

In the first year of treatment, the mean RTSS scores for the 300IR and 500IR doses were statistically significantly reduced compared with placebo from the first day of the pollen season. This effect continued during the pollen season. A statistically significant improvement was also observed throughout the peak of the first pollen season.

Additional analyses were performed on the ITT population, and the data for the ancova of the average RTSS, with treatment and pooled sites as factors and retrospective RTSS as covariate, showed a highly statistically significant difference between the 100IR group compared with the 300IR group and no significant difference between the 300IR group and the 500IR group (*P*=0.0015 and 0.6082, respectively). For comparisons of the primary efficacy variable in the active groups vs. the placebo group using only the 10 pooled sites with the highest pollen count, analyses showed statistically significant differences between the 300IR and 500IR groups, compared with the placebo group (*P*=0.0025 and 0.0303, respectively) and no statistically significant difference between those treated with 100IR and those on placebo (*P*=0.4591); these data confirm the findings of the primary analyses.

The early treatment effect was also observed when the six median individual rhinoconjunctivitis symptoms were analysed. The individual symptoms improved significantly compared with placebo in the 300IR and 500IR treatment groups for the pollen season. In contrast, there was no significant improvement seen in the 100IR group. In the 300IR group, nearly all individual symptoms improved during the pollen season compared with placebo (median improvement of 13% sneezing, 25% runny nose, 33% itchy nose, 59% nasal congestion, 50% watery eyes and 30% itchy eyes). The most marked improvements observed for a median individual symptom score were for nasal congestion and eye symptoms (watery eyes, itchy eyes). Efficacy in each symptom leads to overall improvement in RQLQ.

**Figure 5:**
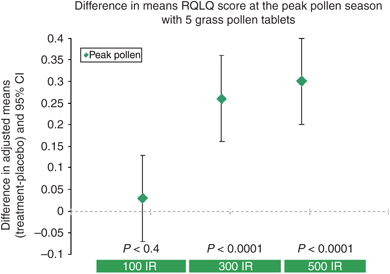
Effect of treatment on quality of life scores recorded during the peak pollen period as measured by Rhinoconjunctivitis Quality of Life Questionnaire (RQLQ).

Compared with subjects in the placebo group, those taking the 300IR and 500IR doses reported significantly improved quality of life using the mean RQLQ scores during the peak pollen season (*P*<0.0001 and <0.0001, respectively) (Fig. 5). These results were also confirmed at the end of the pollen season (*P*=0.0031 and <0.0001, respectively).

Treatment as assessed by subjects themselves (subjects' global evaluation, where they compared the pollen season in the first year of treatment with the previous year's pollen season) was statistically significantly effective only for subjects receiving the 300IR (*P*=0.0001) and 500IR doses (*P*<0.0001) compared with placebo.

During the peak of the first pollen season after treatment initiation, the proportion of days subjects needed rescue medication was significantly less in the groups receiving 300IR (*P*=0.042) and 500IR (*P*=0.015) compared with those on placebo. For the entire pollen season, the proportion of days on any rescue medication was significantly less in the 300IR group compared with the placebo group (*P*=0.019).

### Safety

All treatment doses were well tolerated. Adverse events were particularly common during the dose escalation phase (the first 5 days after treatment initiation). The local events, such as mild itching and swelling of the floor of the mouth, were common. Between 1.9% and 6.4% of the patients reported severe adverse events, usually oral pruritus or, more rarely, gastrointestinal pain. A few of them required treatment. The duration of events was highly variable; the median duration of oral pruritus was 1, 12, 12.5 and 5 days for patients taking placebo, 100IR, 300IR and 500IR, respectively.

In this current study, the systemic reactions possibly, probably and certainly related to the treatment included eight episodes of rhinitis (two in the 100IR group, four in the 300IR group and two in the 500IR group) and two episodes of urticaria in the 100IR group. No serious systemic events were observed.

### Immunological markers

In the first year after treatment initiation, the specific IgE (kU/L) levels increased by a factor of 2.0 or more for all treatment groups, while for the placebo group, the geometric means remained similar at the two visits (a ratio of 1.0). Timothy grass IgG4 (mg/L) levels remained static in the placebo group but increased in all treatment groups. These increases in IgE and IgG4 levels (measured at the end of the first pollen season) were highly significant in all treatment groups, compared with placebo (*P*<0.0001).

## Discussion

The safety and efficacy of this five-grass pollen SLIT tablet has been presented previously and showed that the 300IR and 500IR doses, but not the 100IR dose, were significantly effective compared with the placebo. The benefit-to-risk ratio identified the 300IR tablet as the optimal dose of allergen [9].

The data in the current paper shows that the initiation of treatment effect began in the first pollen season after a 4-month pre-seasonal treatment. In comparison with placebo, almost all efficacy measures achieved significantly improved outcomes in subjects taking the optimal 300IR dose in this first treatment season. These doses achieved a statistically significant reduction in both symptom and medication scores compared with placebo, together with a significant improvement in quality of life, which is also supported by the subjects' global evaluation.

Patients throughout Europe are concomitantly exposed to multiple pollens from distinct Pooideae species. Given the overlap in pollination calendars and similar grain morphology, it is not possible to identify which grass species are present in the environment from pollen counts. Furthermore, neither serum IgE reactivity nor skin prick testing allows us to identify which grass species are involved in patient sensitization. Because of their high level of amino acid sequence homology (e.g. >90% for group 1, 55–80% for group 5), a significant cross-immunogenicity is observed between allergens from Pooideae pollens. Nevertheless, pollen allergens also contain species-specific T or B cell epitopes, and substantial quantitative differences exist in allergen (e.g. group 1 and 5) composition between pollens from distinct grass species.

In this context, a mixture of pollens from common and well-characterized Pooideae such as *A. odoratum, D. glomerata, L. perenne, P. pratense* and *P. pratensis* is suitable for immunotherapy purposes because: it is validated, both in terms of safety and efficacy, by established clinical practice; it reflects natural exposure and sensitization conditions (and it ensures a consistent and well-balanced composition of critical allergens, thus extending the repertoire of T and B cell epitopes present in the vaccine) [12].

These data are of clinical and practical significance because immunotherapy is the only treatment for allergic rhinitis with the potential for long-term effect. Nevertheless, current treatment protocols require an initiation phase before the onset of the pollen season. Indeed, studies with other immunotherapy products for the treatment of grass pollen-induced allergic rhinitis have found that treatment must begin 4 months before the pollen season for maximal effect and at least 8 weeks before the season to provide any evidence of efficacy [8, 14].

Data from one of the studies with grass pollen tablet immunotherapy suggested that the duration of the pre-seasonal treatment period may influence the magnitude of the clinical effect [12, 14]. In this current study, the treatment was started 4 months before the pollen season. Further investigations are needed to determine the optimal duration of the pre-treatment phase to obtain immediate- and long-term efficacy with grass pollen SLIT.

In our study, we clearly showed that a 4-month pre-seasonal treatment resulted in efficacy in the first pollen season. The rationale for initiating treatment 4 months before the expected start of the season is to allow time for the activation of suppressor T cells, regulate the Th1/Th2 cytokine balance and shift it towards the Th1 cells, which relieves hypersensitivity to the target allergen. Our results demonstrate that IgG4 increases in the first pollen season, suggesting that the immunological changes necessary to relieve hypersensitivity have occurred before the end of the first pollen season. This finding supports the efficacy data observed in this study.

## Conclusions

In conclusion, it could be demonstrated that multi-centre trials in pollen-allergic patients need to pay special attention to the variation in the pollen season in Europe. The clinical efficacy of SLIT with grass allergen tablets was demonstrated in both the 300IR and 500IR treatment groups in the regional pollen seasons. RTSS was significantly lower (vs. placebo) during the local pollen season and during the peak pollen period with both 300IR and 500IR tablets. Rhinitis quality of life scoring was significantly better (vs. placebo) during local peak pollen periods and at end of pollen period using either 300IR or 500IR tablets. No significant differences were obtained using tablets with only 100IR.

We were able to demonstrate the efficacy of a five-grass pollen SLIT tablet from the first season with a 4-month pre-seasonal treatment phase. These results confirm the conclusions of the primary evaluations and validate the use of 300IR tablets for clinical practice.
